# Supplementary material for: Using a synthetic machinery to improve carbon yield with acetylphosphate as the core
Source: Nat Commun. 2023 Aug 30;14:5286. doi: 10.1038/s41467-023-41135-7 (PMC10468489; doi:10.1038/s41467-023-41135-7)
Supplement: Supplementary file 1 — Supplementary Information [file 41467_2023_41135_MOESM1_ESM.pdf]

Using a synthetic machinery to improve carbon yield with acetylphosphate as the core

Guo *et al.*

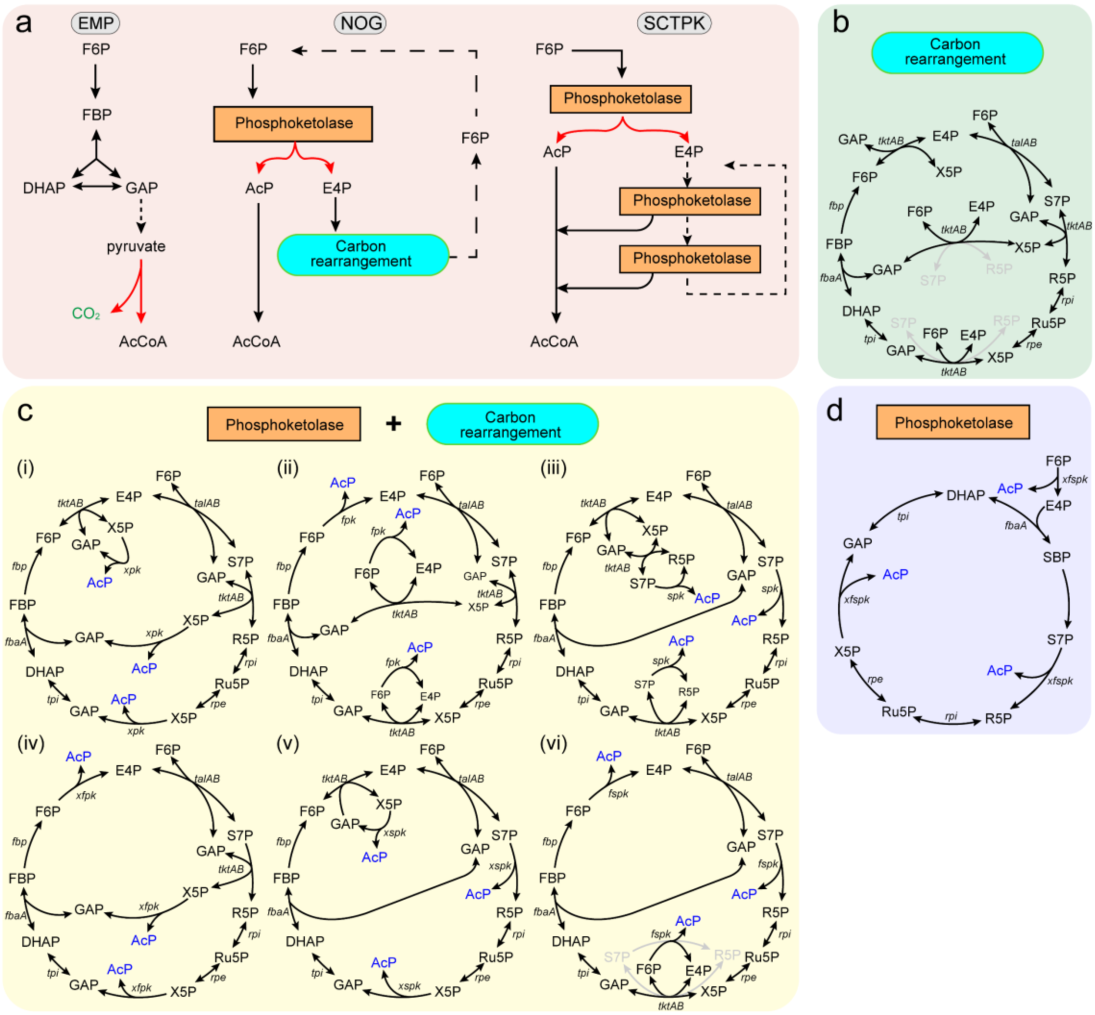

Supplementary Figure 1. Structure of all networks based on phosphoketolase and carbon rearrangement. a. Simplified schematic of EMP, NOG and SCTPK. The most common metabolic route for AcCoA synthesis in *E. coli* is the glycolysis coupled with the release of two mol of CO_2 per mol of glucose, which lowers the atomic economy of targeted chemical biosynthetic pathway. The original NOG[1] includes phosphoketolase and carbon rearrangement steps, bypassing the CO_2 release process. The SCTPK includes only the catalytic step of phosphoketolase, does not require promiscuous carbon rearrangement reactions while achieving no CO_2 release. **b**. Natural pathways in *E. coli*. When there is no phosphoketolase but only carbon rearrangement mediated by transketolase and transaldolase, there is no direct generation of AcP. Transketolase encoded by *tktAB* catalyzes reactions: F6P + GAP <=> E4P + X5P, R5P + X5P <=> S7P + GAP; Transaldolase encoded by *talAB* catalyzes reactions: F6P + E4P <=> S7P +

GAP. **c**. All six configurations of the pattern of "phosphoketolase + carbon rearrangement". Phosphoketolase with different activities for three sugar phosphates (X5P, F6P and S7P) replaces the carbon rearrangement in these steps to provide AcP, and then F6P was supplemented by the carbon rearrangement circularly. (i) Phosphoketolase cleaves X5P to generate GAP and AcP, which replaces the reactions catalyzed by transketolase "X5P + R5P <=> S7P + GAP, X5P + E4P <=> F6P + GAP; (ii) Phosphoketolase cleaves F6P to generate E4P and AcP, which replaces the reaction catalyzed by transketolase "F6P + GAP <=> E4P + X5P"; (iii) Phosphoketolase cleaves S7P to generate R5P and AcP, which replaces the reactions catalyzed by transketolase and transaldolase "S7P + GAP <=> X5P + R5P, S7P + GAP <=> F6P + E4P"; (iv) NOG structure [1]. Phosphoketolase cleaves F6P and X5P to generate R5P, AcP and E4P, AcP, respectively. (v) Phosphoketolase cleaves X5P and S7P to generate GAP, AcP and R5P, AcP, respectively. (vi) Phosphoketolase cleaves F6P and S7P to generate E4P, AcP and R5P, AcP, respectively. **d**. The configuration of the pattern of "phosphoketolase". Phosphoketolase cleaves X5P, F6P and S7P to provide AcP. Carbon rearrangement is no longer required to maintain circulation and can be excised to prevent interference from other pathways. Abbreviations: F6P, fructose-6-phosphate; GAP, glyceraldehyde-3-phosphate; DHAP, dihydroxyacetone phosphate; AcP, acetylphosphate; G6P, glucose-6-phosphate; F6P, fructose-6-phosphate; FBP, fructose-1,6-biphosphate; AcCoA, acetyl-CoA; S7P, sedoheptulose-7-phosphate; X5P, xylulose-5-phosphate; E4P, erythrose-4-phosphate; R5P, ribose-5-phosphate; Ru5P, ribulose-5-phosphae.

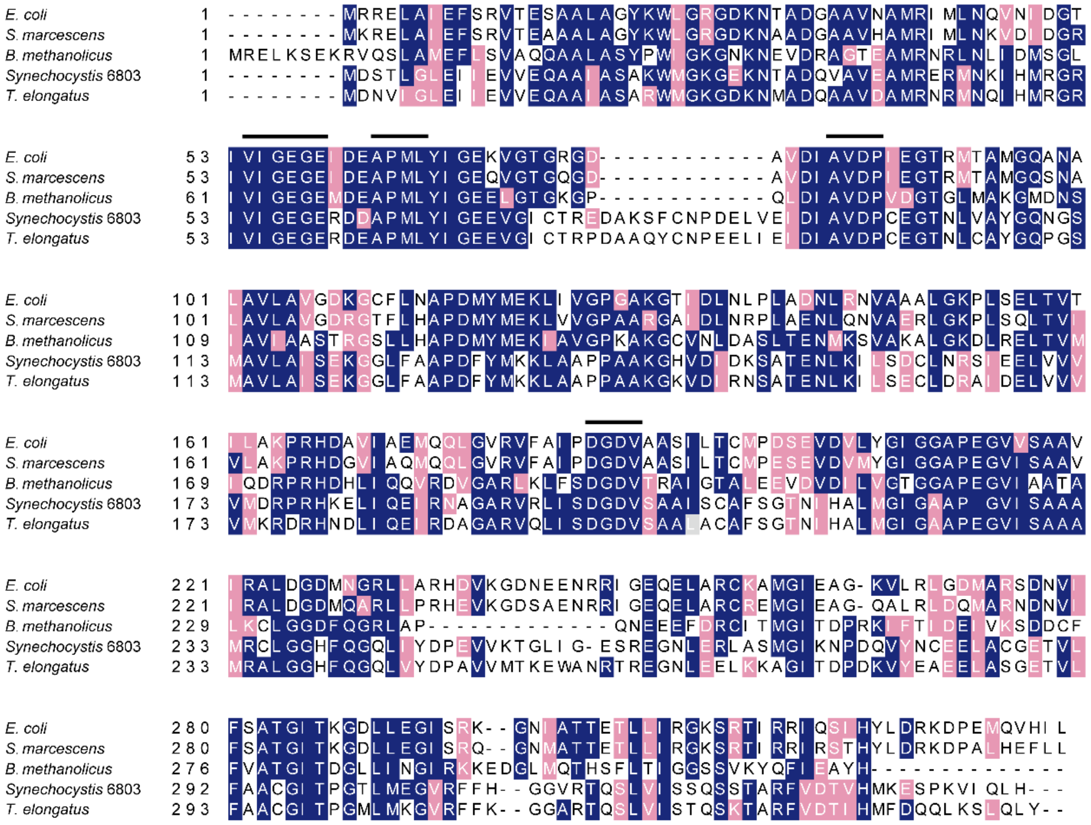

Supplementary Figure 2. Sequence alignment between selected prokaryotic-derived FBPases whose hosts were *Escherichia coli* BW25113, *Serratia marcescens*, *Bacillus methanolicus*, *Synechocystis* sp. PCC6803 and *Thermosynechococcus elongatus*. Dark blue boxes indicate identical residues and light pink ones indicate conservative substitutions. Alignments were generated with Clustal Omega (https://www.ebi.ac.uk/Tools/msa/clustalo) and formatted with Boxshade (https://github.com/pinbo/boxshade). The conserved domains are indicated by black lines and considered to be the binding site of Li^+ [2, 3].

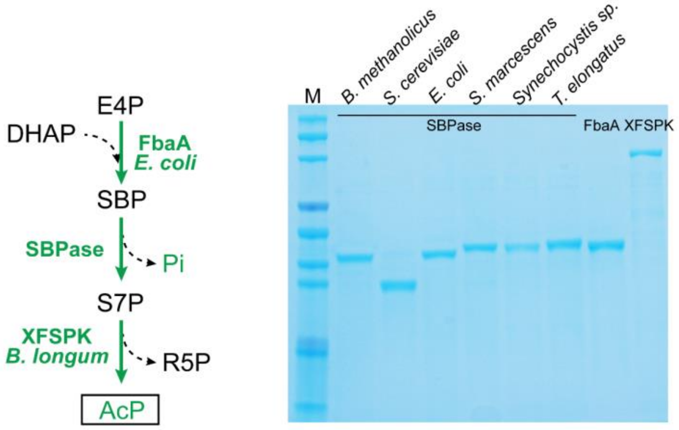

Supplementary Figure 3. SDS-PAGE of purified related enzymes associated with *in vitro* cascade reactions. These enzymes include fructose bisphosphate aldolase FbaA from *E. coli*, phosphoketolase XFSPK from *B. longum* and SBPases from six different strains (*Bacillus methanolicus*, *Saccharomyces cerevisiae* CEN.PK, *Escherichia coli* BW25113, *Serratia marcescens*, *Synechocystis* sp. PCC 6803, *Thermosynechococcus elongatus*). Source data are provided as a Source Data file.

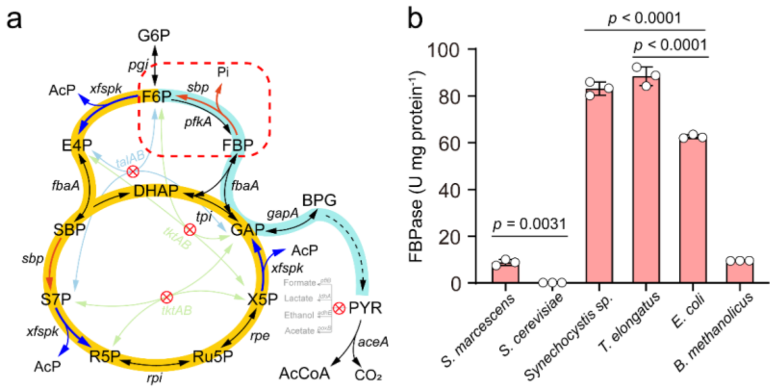

Supplementary Figure 4. FBPase enzyme activity assay. a. FBPase encoded by *sbp* catalyzes the production of F6P from FBP, which is the first enzyme linking the glycolytic to the carbon conversion pathway SCTPK. **b**. Comparison of FBPase enzyme activities of different hosts. All data were the average of three independent studies with standard deviations. Two-tailed Student's *t* tests were performed to determine the statistical significance for two group comparisons. Source data are provided as a Source Data file.

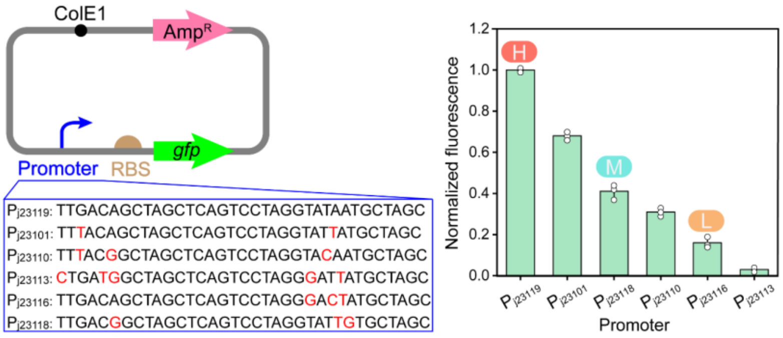

Supplementary Figure 5. Schematic of assay used to test the strength of the promoters. Amp, *β*-lactamase gene. Determination of the strengths of the six promoters in LB. P_{j23119}, P_{j23118}, P_{j23116} were selected as strong (H), medium (M) and weak promoter (L), respectively. All data were the average of three independent studies with standard deviations. Source data are provided as a Source Data file.

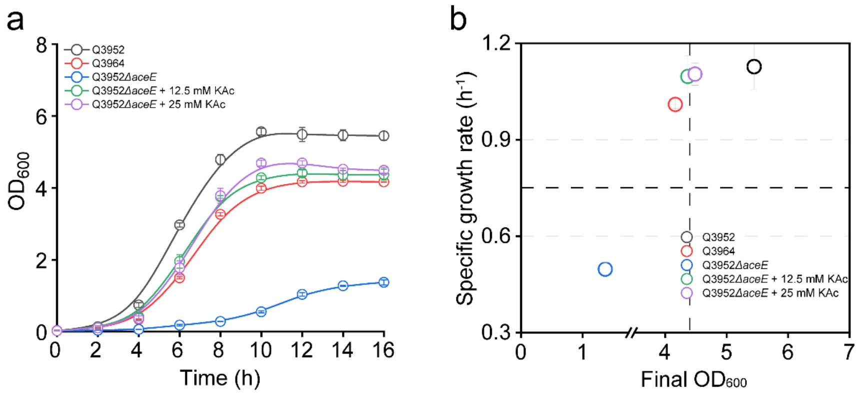

Supplementary Figure 6. The growth status of strains in LB medium was used for *in vivo* validation of the constructed SCTPK. a. Validation of whether the SCTPK could complement AcCoA by strain growth detection. The OD_{600} of different types of strains were detected, including those assembling the SCTPK pathway, *aceE* knockout strains and strains further supplemented with potassium acetate (KAc). **b**. Specific growth rates and final OD_{600} of the above strains. All data were the average of three independent studies with standard deviations. Source data are provided as a Source Data file.

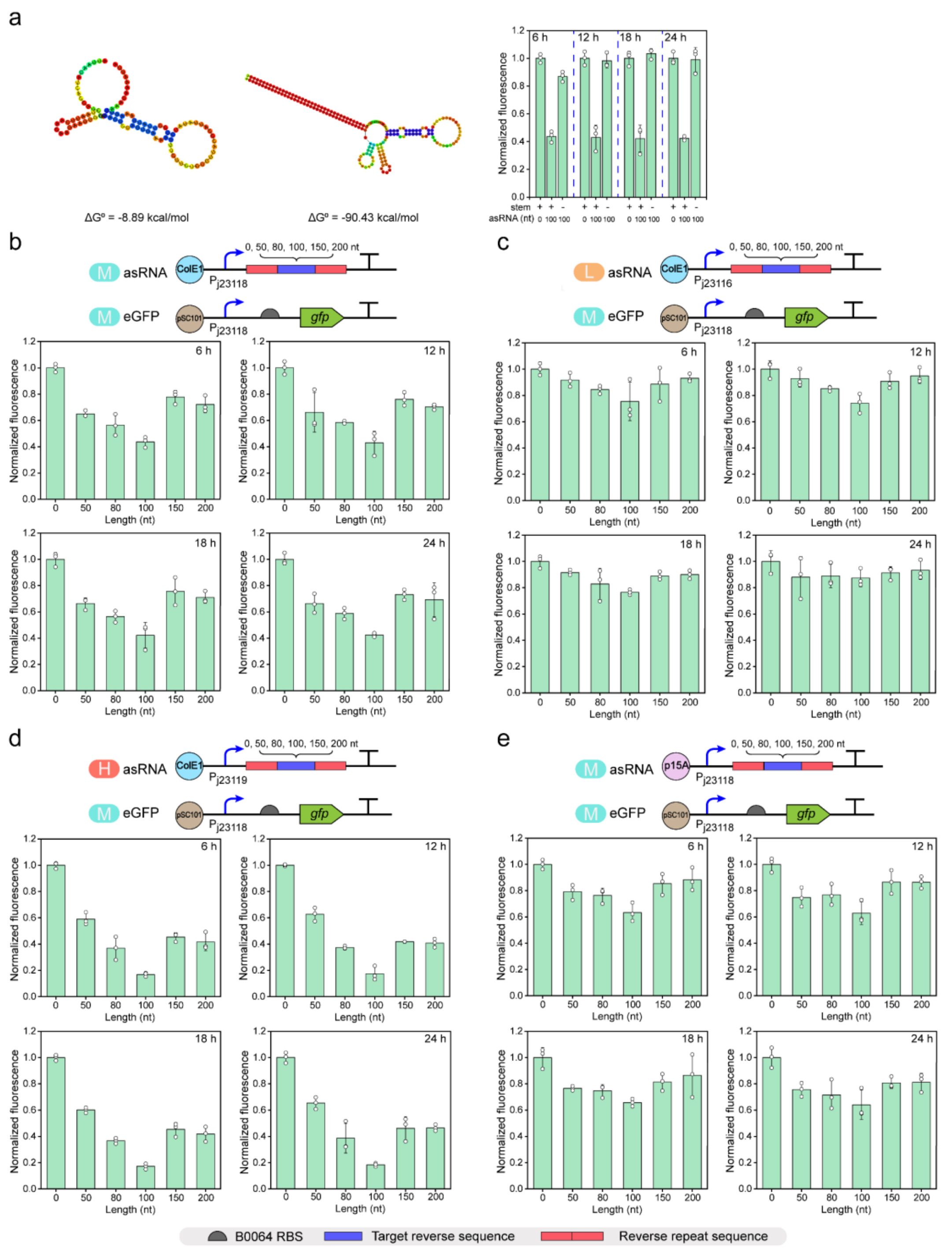

Supplementary Figure 7. Screening and determining the effect of different bioparts on antisense RNA repression properties. a. The left panel shows the simulated secondary structures of 100 nt target asRNA with (right) or without (left) the stem. The structure predictions and their corresponding Gibbs free energy calculations were performed using a web-based prediction tool, RNAfold (http://rna.tbi.univie.ac.at//cgibin/RNAWebSuite-/RNAfold). The right panel displays

the comparison of fluorescence with or without the stem. The expression of asRNA was set on the plasmid with ColE1 replication origin, while the target gene *gfp* was on the plasmid with pSC101. The promoters were all P_{j23118}. **b**. Effect of target sequence length on asRNA interference efficiency. ColE1, pSC101, replication origin. The expression of asRNA was set on the plasmid with ColE1 origin, and the promoters were P_{j23118}. **c**. **d**. Hierarchical interference efficiency through regulation of the transcription of the asRNA by different promoters. The promoter of asRNA was replaced by weak promoter P_{j23116} (**c**) or strong promoter P_{j23119} (**d**). **e**. The influence of replication origin of plasmid expressing asRNA on interference efficiency. The replication origin for asRNA expression was replaced by p15A with medium copy number. The promoters of asRNA and *gfp* were P_{j23118}. The relative fluorescence intensity of the strains was detected at 6, 12, 18 and 24 h, respectively, and compared with those containing the stem and the target length of 0 bp. All data were the average of three independent studies with standard deviations. Source data are provided as a Source Data file.

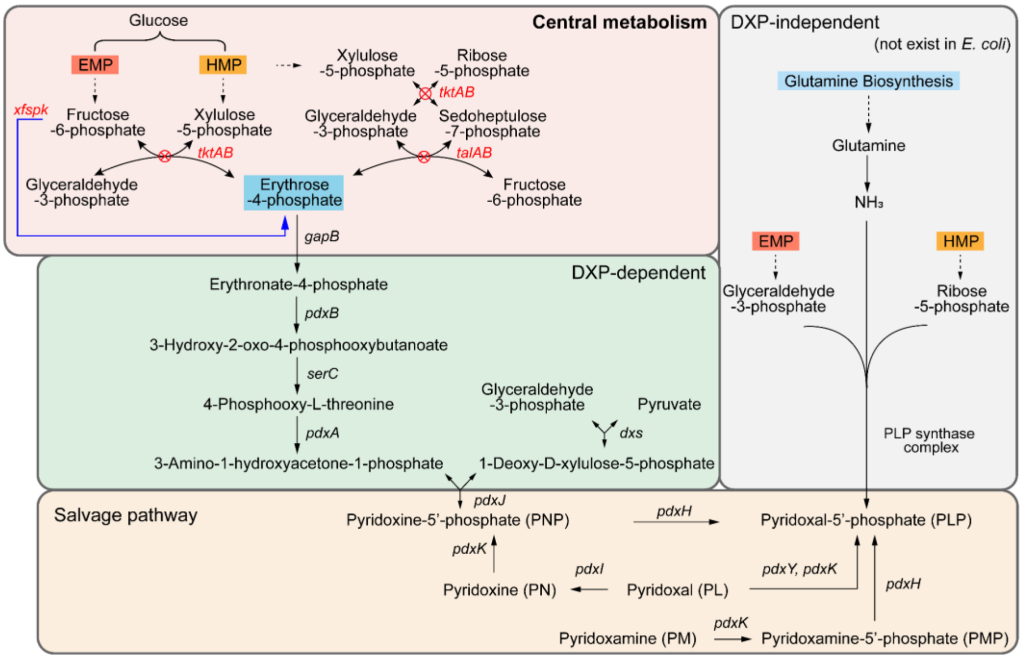

Supplementary Figure 8. Biological function of *gapB*. Biosynthesis of vitamin B6 includes 1-deoxy-D-xylulose-5-phosphate (DXP)-dependent, salvage and DXP-independent pathways. However, only the first two pathways exist in *E. coli*. The vitamin B6 comprises a group of the six B6 vitamers pyridoxine (PN), pyridoxal (PL), pyridoxamine (PM), and their respective 5'-phosphate esters pyridoxine 5'-phosphate (PNP), pyridoxal 5'-phosphate (PLP), and pyridoxamine 5'-phosphate (PMP). PLP is a cofactor for more than 185 enzymatic reactions (http://bioinformatics.unipr.it/cgibin/bioinformatics/B6db/h- ome.pl). Most of these enzymes are involved in amino acid metabolism. Carbon rearrangement enzymes transketolase and transaldolase encoded by *tktAB* and *talAB* catalyze the production of erythrose-4-phosphate (E4P). After inactivation of *tktAB* and *talAB* and integration of the SCTPK, the strain could grow in the absence of exogenous additive (Fig. 2d, f); asRNA inhibition of *gapB* impaired the growth of the strain (Fig. 3d), indirectly demonstrating that the introduced Xfspk possesses the cleaving activity of hexose F6P. In the DXP-dependent pathway, the erythrose-4-phosphate dehydrogenase encoded by *gapB* uses E4P as substrate to generate erythronate-4-phosphate, which is an inhibitor of ribose-5-phosphate isomerase (RpI)[4], and then gradually converts into 3-amino-1-hydroxyacetone-1-phosphate. Finally, it is condensed with DXP by the PNP synthase (PdxJ) to provide PNP. Therefore, *gapB* is the key gene that shunts E4P into vitamin B6.

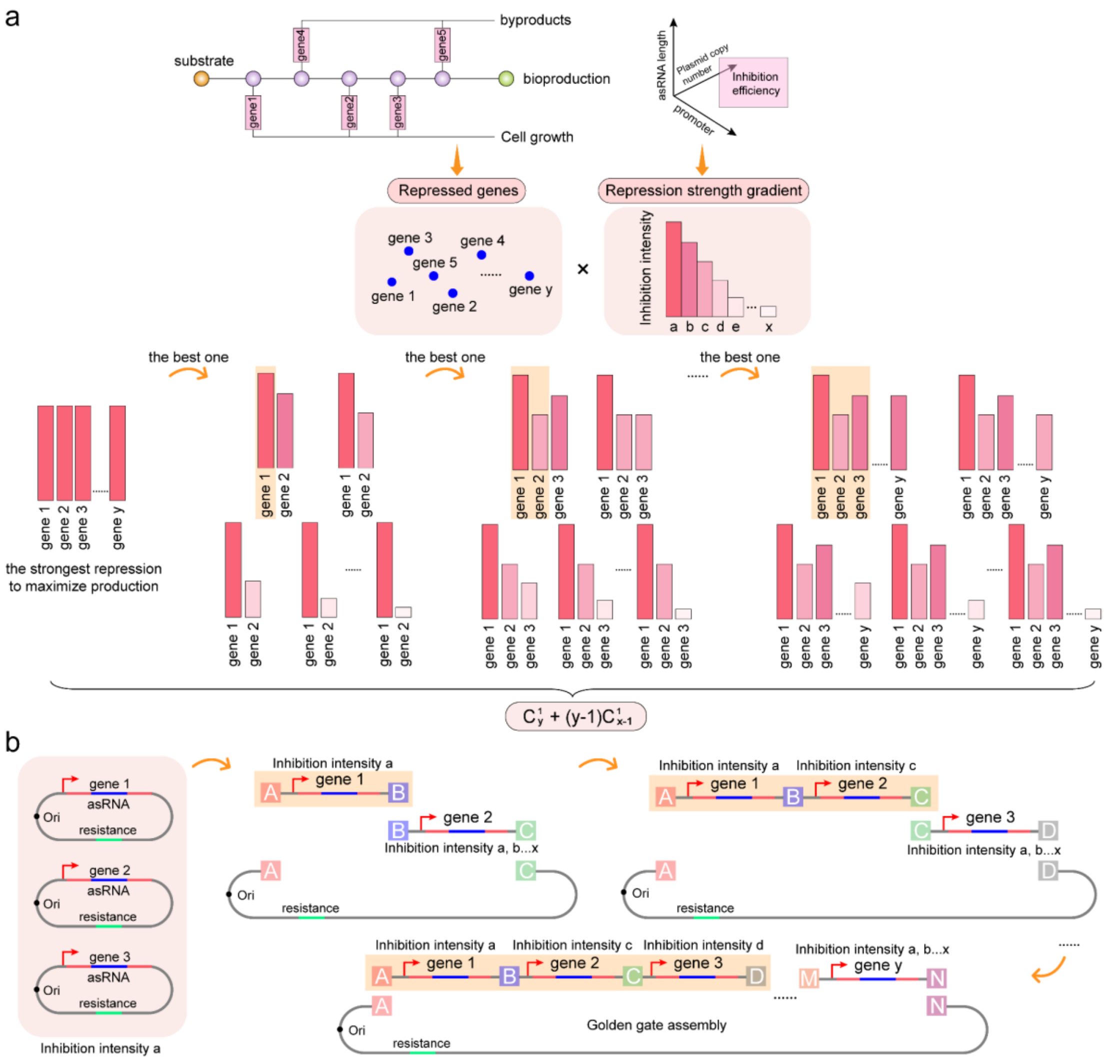

Supplementary Figure 9. Combination and optimization of multiple gene repression by hierarchical regulation of asRNA arrays. a. Patterns of asRNA gradient repression**.** In order to maximize the metabolic flux into the chemicals synthesis route, competing metabolic pathways, including those related to byproducts or cell growth, are often inhibited. By adjusting the length of asRNA and controlling the transcription of it (Supplementary Fig. 7), different inhibition intensities could be obtained, and thus the target genes could be repressed in a hierarchical manner. In the first round, the strongest repression needs to be selected on individual genes to allow for maximizing the synthesis of the target product. Select the strain with the highest production of the target product without affecting the basic growth to continue the next round of combinatorial inhibition. One target gene was evaluated in each round in turn

for the remaining gradient of inhibition intensity, and the strain with the highest production while satisfying basic growth was selected for the next round of inhibition. Therefore if there are x repressive intensities and hierarchical regulation of y genes is required, there are no more than $\left[C_y^1 + (y-1)C_{x-1}^1\right]$ combinations in total. **b**. Construction of asRNA arrays with different inhibition strengths to gradually screen the strains with the highest production of target chemicals while meeting basic growth by Golden gate assembly. Due to the presence of homologous sequences, Golden gate assembly is more advantageous than Gibson assembly. ColE1, replication origin; Amp, β-lactamase gene. Background fonts A, B, C, D are the sticky ends of type IIs endonuclease Eco31I.

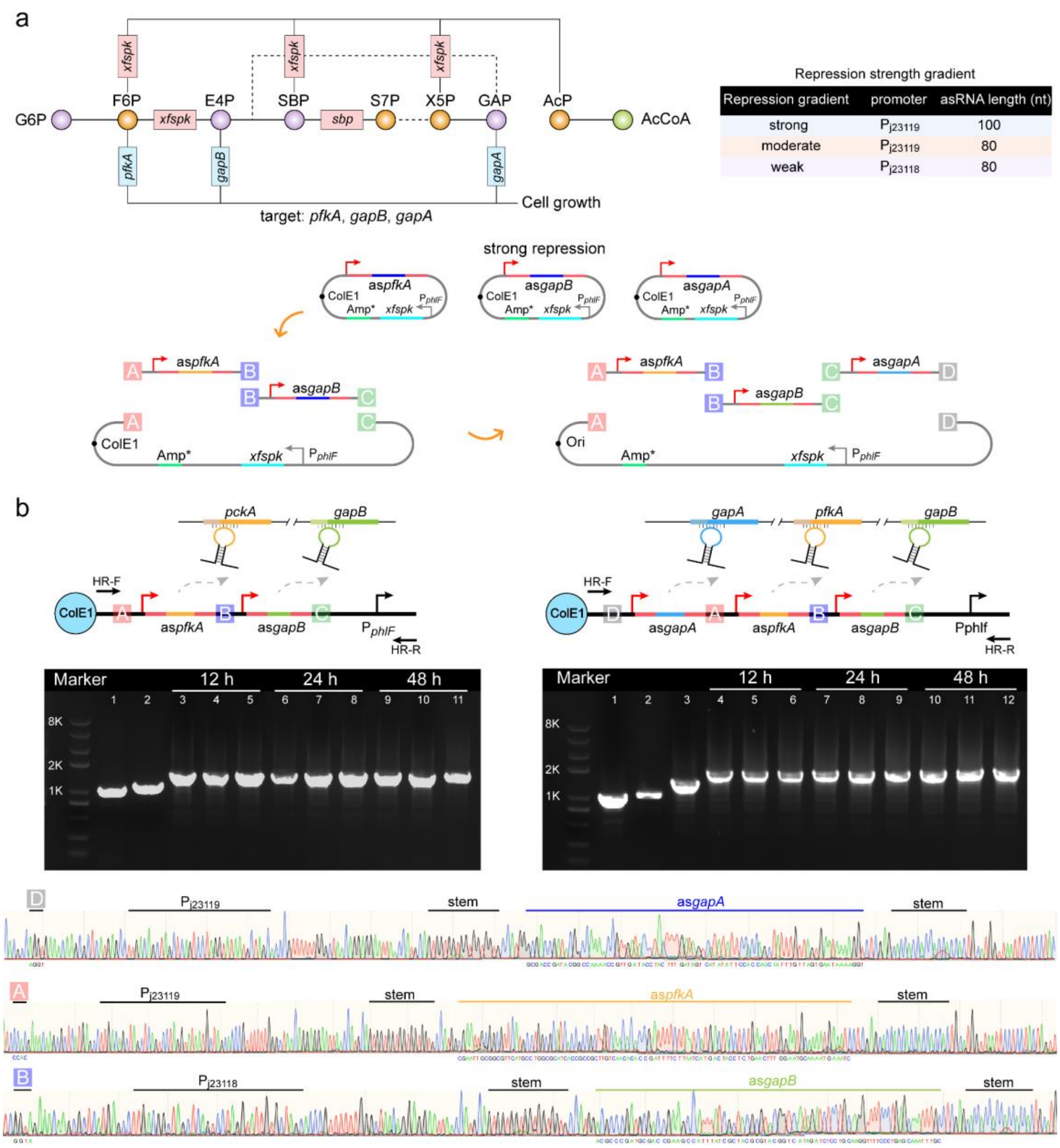

Supplementary Figure 10. Hierarchical regulation of SCTPK competitive pathway by combinatorial asRNA arrays. a. asRNAs with different repression strength chosen for the essential genes (*pfkA*, *gapB* and *gapA*) controlling three major competitive pathways on AcP production. In the first round, the three genes were strongly repressed to evaluate the strain with the best fermentation performance, followed by a combination of hierarchical repression of the other genes. **b**. Stability of constitutive asRNA arrays expression. Images of gels post electrophoresis showed the genetic stability of asRNA arrays expression plasmid under double, or triple silence co-existing conditions The left panel displayed the stability of plasmids containing double asRNAs silencing tested at different times using the indicated primers. Lane 1:

containing stem without target asRNA sequence; Lane 2: containing stem + as*pfkA*(100nt, P_{j23119} promoter); Lane 3, 6, 9: containing stem + as*pfkA*(100 nt, P_{j23119} promoter), and stem + as*gapB*(100 nt, P_{j23119} promoter); Lane 4, 7, 10: containing stem + as*pfkA*(100 nt, P_{j23119} promoter), and stem + as*gapB*(80nt, P_{j23119} promoter); Lane 5, 8, 11: containing stem + as*pfkA*(100 nt, P_{j23119} promoter) and stem + as*gapB*(80nt, P_{j23118} promoter). The right panel presented the stability of plasmids containing triple asRNAs silencing tested at different times using the indicated primers. Lane 1: containing stem without target asRNA sequence; Lane 2: containing stem + as*pfkA*(100nt, P_{j23119} promoter); Lane 3: containing stem + as*pfkA*(100nt, P_{j23119} promoter), and stem + as*gapB*(80nt, P_{j23118} promoter); Lane 4, 7, 10: containing stem + as*pfkA*(100nt, P_{j23119} promoter), stem + as*gapB*(80nt, P_{j23118} promoter), stem + as*gapA*(80nt, P_{j23119} promoter); Lane 5, 8, 11: containing stem + as*pfkA*(100nt, P_{j23119} promoter), stem + as*gapB*(80nt, P_{j23118} promoter), stem + as*gapA*(80nt, P_{j23118} promoter); Lane 6, 9, 12: containing stem + as*pfkA*(80nt, P_{j23119} promoter), stem + as*gapB*(80nt, P_{j23119} promoter), stem + as*gapA*(80nt, P_{j23119} promoter). The 48-hour PCR product of lane 10 was sequenced and no base mutations were found. Source data are provided as a Source Data file.

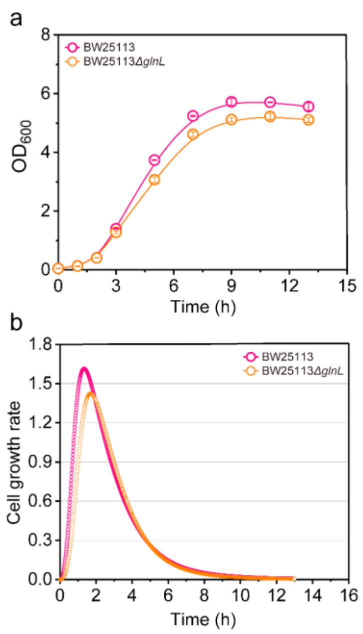

Supplementary Figure 11. Growth profile of the strain with *glnL* knockout. a. **c**. Growth curves of control (wild) and *glnL* knockout strains in (**a**) LB (10 g/L tryptone, 5 g/L yeast extract and 10 g/L sodium chloride) and (**c**) M9 modified minimal medium (14 g/L $K_2HPO_4{\cdot}3H_2O$, 5.2 g/L KH_2PO_4, 1 g/L NaCl, 1 g/L NH_4Cl, 0.25 g/L $MgSO_4$ without addition of yeast extract). **b**. **d**. Cell growth rates of control (wild) and *glnL* knockout strains in (**b**) LB and (**d**) M9 modified minimal medium. All data were the average of three independent studies with standard deviations. Source data are provided as a Source Data file.

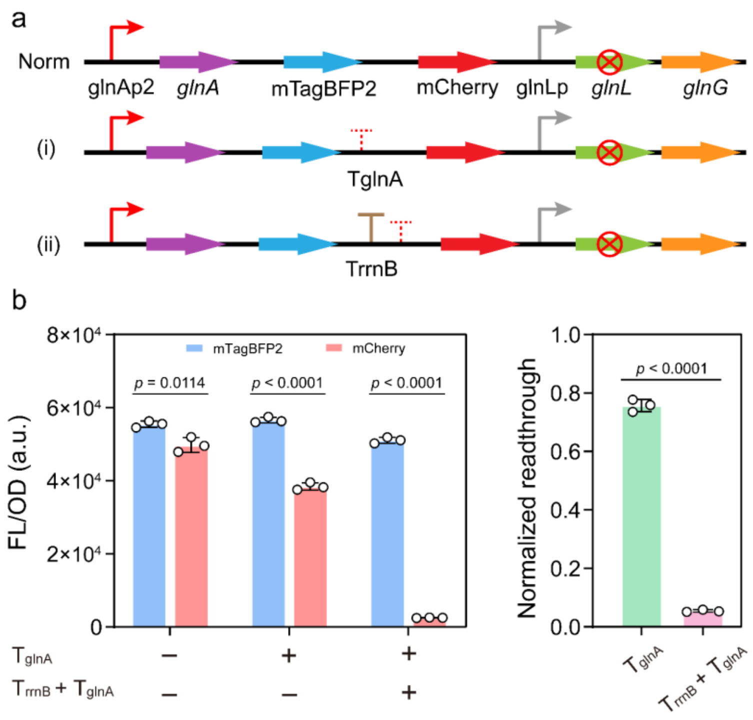

Supplementary Figure 12. Modification of *glnALG* operon and elimination of the feedback loop. a. The genome was modified to introduce double fluorescent proteins and a terminator rrnb T1 into the original *glnALG* operon to obtain strains Norm, (i) and (ii). Specifically, the *glnA* terminator was removed in the Norm strain, and fluorescent proteins mTagBFP2 and mCherry were introduced before and after the original *glnA* terminator position. The *glnA* terminator was not removed from strain (i), and the fluorescent proteins were inserted into the same position as Norm. In strain (ii), in addition to the fluorescent proteins, a strong terminator rrnb T1 was introduced in front of *glnA* terminator. The cysteine located at position -4 of promoter *glnLP* was mutated to thymine to reduce the ability of NRI to block the initiation of transcription[5]. **b**. Determination of the readthrough. The readthrough coefficients ε of (i) and (ii) structures were calculated by measuring the mTagBFP2 and mCherry fluorescence intensities: $\varepsilon_{Norm} = \frac{mCherry_0}{mTagBFP2_0}$, $\varepsilon = \frac{mCherry}{mTagBFP2} \times \frac{1}{\varepsilon_{Norm}}$. All data were the average of three independent studies with standard deviations. Two-tailed Student's *t* tests were performed to determine the statistical significance for two group comparisons. Source data are provided as a Source Data file.

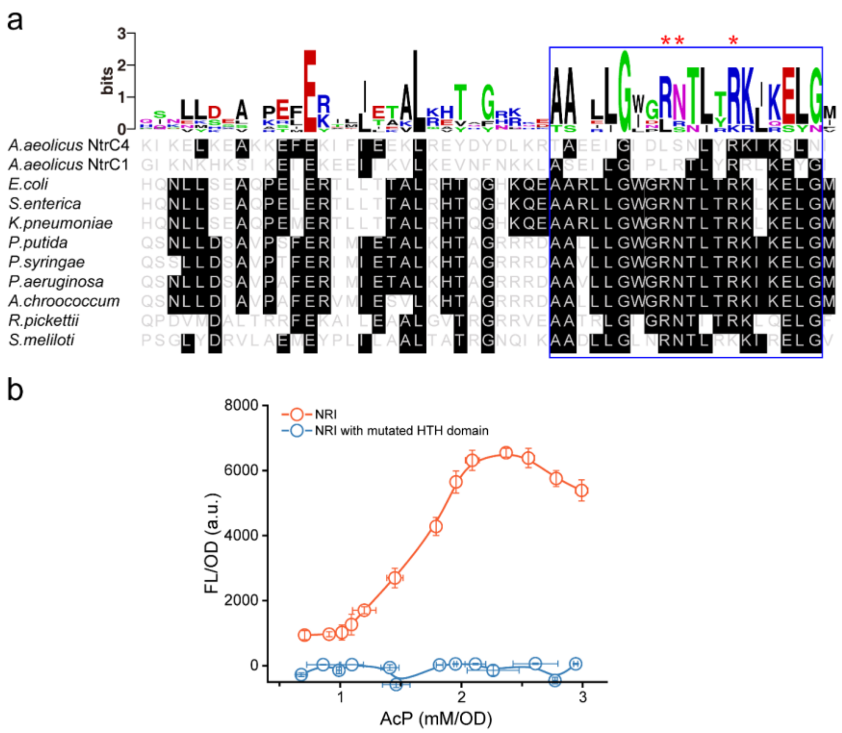

Supplementary Figure 13. Search and verification of NRI helix-to-helix region of *Escherichia coli*. a. Sequence logos of alignment results of NRI (NtrC). The C-terminal domain of *E. coli* BW25113 NRI was aligned with other 10 NRIs which were from *Pseudomonas aeruginosa*, *Sinorhizobium meliloti*, *Azotobacter chroococcum*, *Aquifex aeolicus* NtrC4, *Aquifex aeolicus* NtrC1, *Pseudomonas putida*, *Ralstonia pickettii*, *Helicobacter pylori*, *Pseudomonas syringae*, *Klebsiella pneumoniae*, and *Salmonella enterica*, respectively. The C-terminal of 11 different NRIs contains a conserved region marked by a blue box, which is presumed to be the helix-turn-helix region. The red asterisks marked are the conserved key amino acids Arg, Asn and Arg in *S. enterica* and *E. coli* NRIs, corresponding to Leu, Ser and Arg in *A. aeolicus* NtrC4, respectively. **b**. The helix-to-helix region of the predicted *E. coli* NRI was mutated using CRISPR. The dose-dependent property of the original NRI and the mutated to AcP was investigated by fluorescence intensity detection. All data were the average of three independent studies with standard deviations. Source data are provided as a Source Data file.

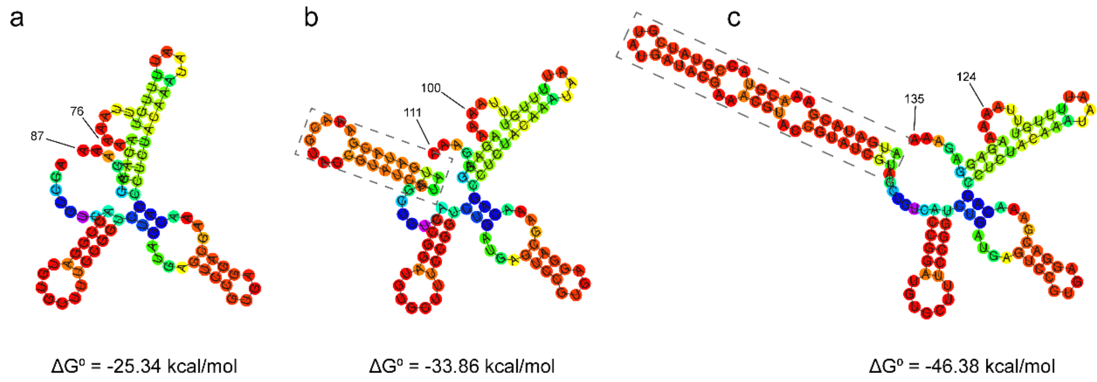

Supplementary Figure 14. The mRNA secondary structures of the 5'UTR region. The 5'UTR region without phlO (**a**), containing 1×phlO (**b**), containing 2×phlO (**c**). The 1×phlO and 2×phlO sites were in the gray dotted box, and the numbered area showed the ribosome-binding site (RBS). The structure predictions and their corresponding Gibbs free energy calculations were performed using a web-based tool, RNAfold (http://rna.tbi.univie.ac.at//cgibin /RNAWebSuite-/RNAfold). Source data are provided as a Source Data file.

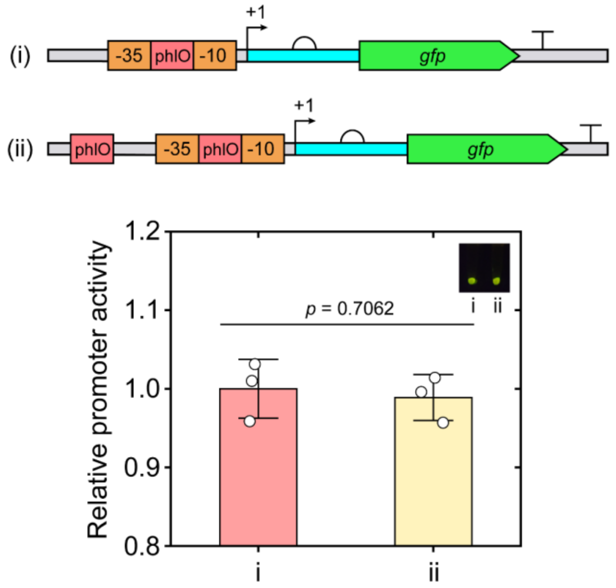

Supplementary Figure 15. Schematic diagram and fluorescence performance of adding the phlO box upstream of the promoter P_{phlF} compared to the original P_{phlF}. The insets were photographs of the two strains, which was taken after centrifugation of 1-mL bacterial solution and irradiation with an LED transilluminator. All data were the average of three independent studies with standard deviations. Two-tailed Student's *t* tests were performed to determine the statistical significance for two group comparisons. Source data are provided as a Source Data file.

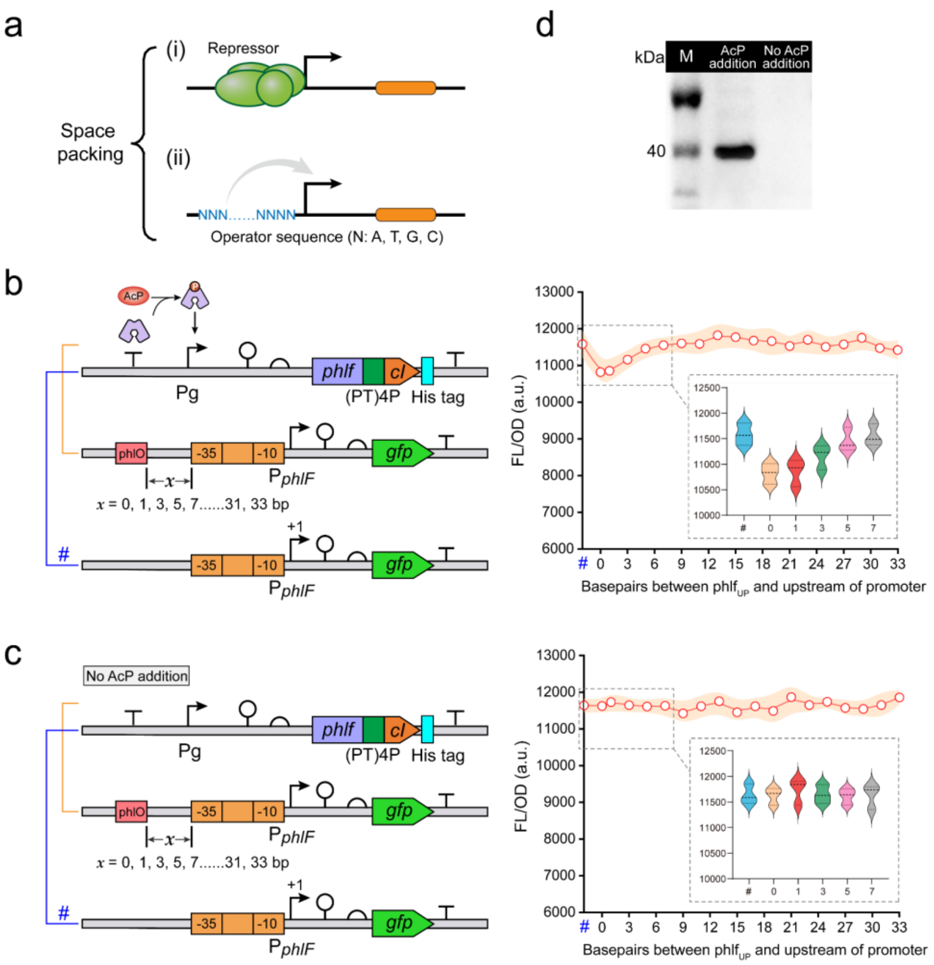

Supplementary Figure 16. Effects of space packing on the promoter activity. a. Classification of space packing**:** (i) The expressed engineered repressor binds to the upstream phlO site, but due to its proximity, the repressor interferes with or interacts with RNA polymerase; (ii) The phlO sequence itself affected the adjacent promoter. **b**. To explore the effect in (i), the spacer between upstream phlO and promoter was set from 0 to 33 bp, 2 bp per step, and AcP was added to detect the fluorescence intensity, while the fluorescence intensity of the system without any phlOs was used as a contrast (#). **c**. To explore the effect in (ii), the genetic circuit was the same as in b, but no AcP was added, and changes in fluorescence intensity were detected, while the fluorescence intensity of the system without any phlOs was used as a contrast (#). **d**. Expression of the engineered repressor was detected by western blot. After the addition of AcP (situation in b), a band with a size of 39.3 kDa was detected, which was consistent with the size of the fused PhlF-(PT)4P-cI, while the corresponding band was not detected without the addition of AcP (situation in c). All data were the average of three independent studies with standard deviations. Source data are provided as a Source Data file.

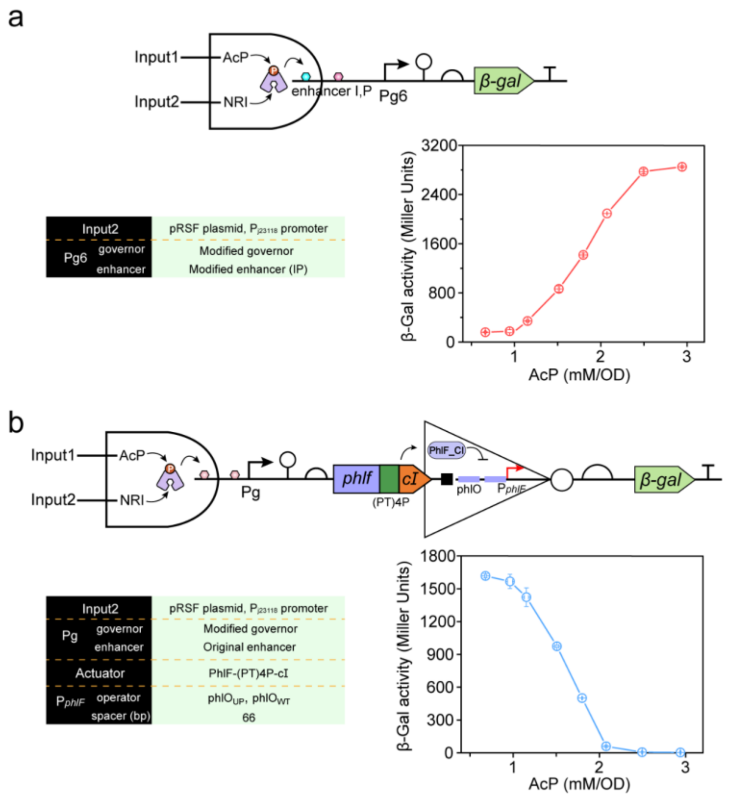

Supplementary Figure 17. The flexibility of regulated genes in the dynamic circuits. The reporter protein GFP was replaced with β-galactosidase (β-gal). Schematic diagram of the dynamic activation (**a**) and repression (**b**) circuits, and characterization of the dynamic activation (**a**) and repression (**b**) of β-galactosidase. All data were the average of three independent studies with standard deviations. Source data are provided as a Source Data file.

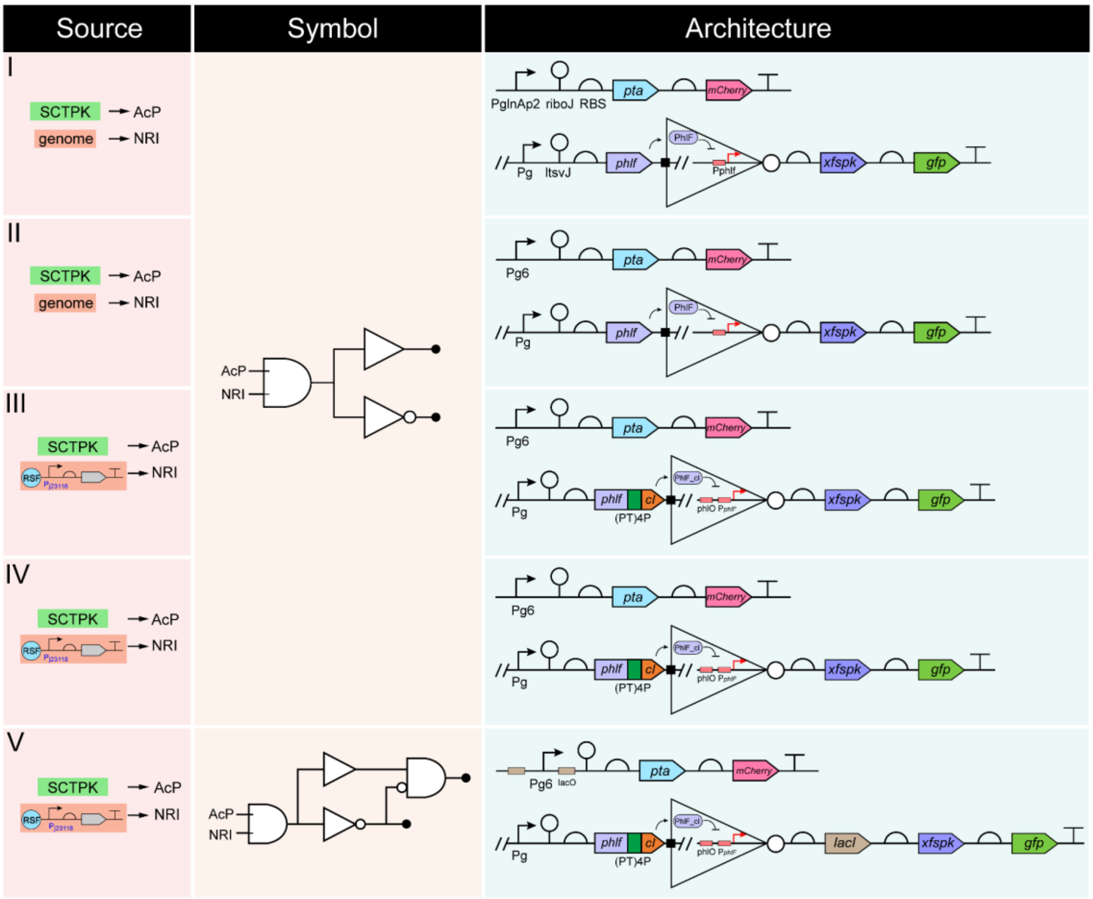

Supplementary Figure 18. Construction of five oscillation devices. Of the two components of the upstream AND gate, AcP was originated from SCTPK after reconfiguration of central metabolism, and the activator protein NRI was sourced from the genomic, RSF replication origin-driven plasmid and under the control of P_{j23116} or P_{j23118}, respectively. The promoters of the downstream activation module include P_{glnAp2} containing the original enhancer, as well as the Pg6 obtained by the mutation and screening (mutated governor, IP enhancer). The downstream repression module includes the original repressor with its paired P_{phlF}, and engineered PhlF-(PT)4P-cI with P_{phlF} containing double operators. The distance between these two operators was 66 bp. In addition, in device V, LacI was added to the output of the downstream repression module and lacO operators were added to the upstream and downstream of Pg6 in the activation module, respectively.

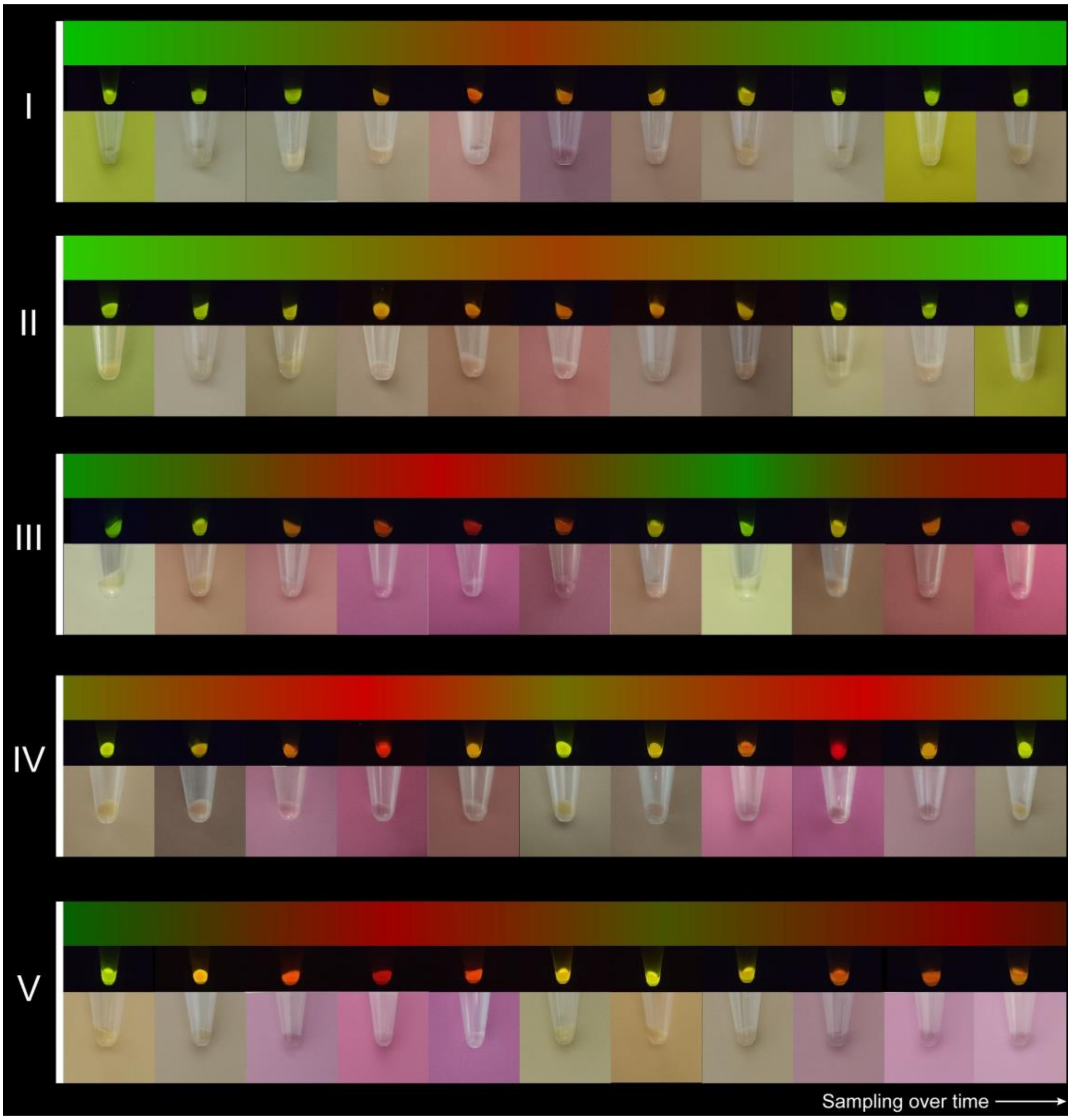

Supplementary Figure 19. Temporal specificity of oscillatory systems for gene expression. *E. coli* carrying different devices were sampled at the same intervals, collected in 1.5-mL tubes and later centrifuged. Fluorescence was taken by a mobile phone with *E. coli* pellets under a LED transilluminator (BL-20, LABGIC). In the bright field, the *E. coli* pellets were also matched to the color chart, and when the color of *E. coli* was basically compatible with the standard, it was taken with a mobile phone. Source data are provided as a Source Data file.

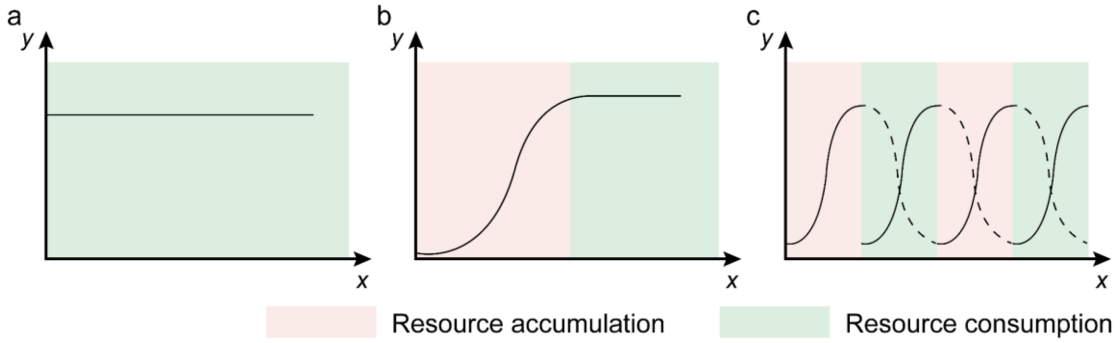

Supplementary Figure 20. Different patterns for regulating metabolic flux. a. Static regulation. In this model, the expression levels of single / multiple genes are generally tuned at a constant level, such as employing promoter or RBS engineering to regulate transcription levels. The most important feature of this modality of modification is directionality, irreversibility. Since the product synthesis related genes are generally overexpressed, the modified cells are in a single state of intracellular resource consumption and do not have the ability to sense specified signals to make specific adjustments. **b**. Dynamic switch. Dynamic switch construction often draws on a sensor, which may be at the single-cell level or at the population level. For the single cell level, such as responding to a key metabolite in the intracellular metabolic pathway, the cascade of signals is transmitted with the help of genetic circuits, which in turn leads to the non-expression of product synthesis genes in the early stage, allowing cells to grow and accumulate intracellular resources. In the later stage, the expression of genes related to product synthesis is turned on and intracellular resources start to be used for the synthesis of the target product, thus achieving the decoupling of growth and production. For the population level, such as quorum sensing, the general mode of construction is that the expression of downstream product synthesis genes is turned on only when the growth of the bacterial reaches a certain level. **c**. Oscillator. Oscillators were proposed in this study as another alternative for the regulation of metabolic flux. Given that the oscillator network possesses an amplitude and periodic expression pattern, when engineered transcriptional regulation and metabolism are integrated to control the generation and consumption pathways of a key metabolite with the help of genetic circuits, its intracellular homeostasis can be effectively maintained, thus eliminating the physiological or biochemical effects caused by excessive expression or consumption. On the other hand, due to its cyclic expression pattern, it may alleviate the waste of carbon resources or even toxicity to cells caused by the excessive accumulation of intermediate metabolites.

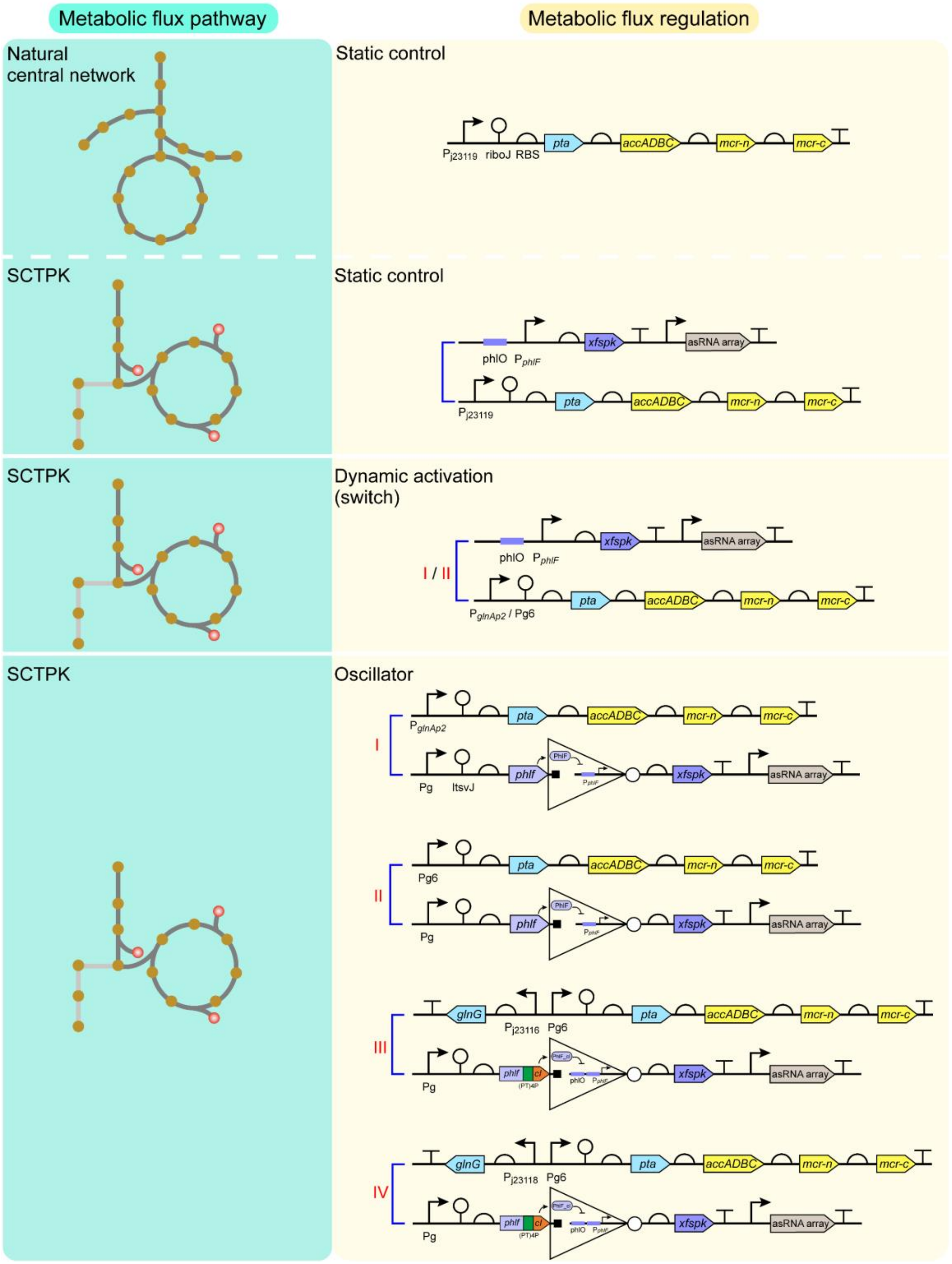

Supplementary Figure 21. Constructing different combinations of regulatory patterns for the biosynthesis of 3HP. Among the metabolic flux pathways include the native central metabolic network and the SCTPK obtained by static reconfiguration. The regulation of metabolic flux includes static control, dynamic activation (switch) and oscillators. Genes related to 3HP synthesis, including *pta*, *accADBC* and *mcr*, were integrated in the downstream activation module. *pta*, phosphate acetyltransferase; *accADBC*, acetyl-CoA carboxylase complex; *mcr*, malonyl-CoA reductase.

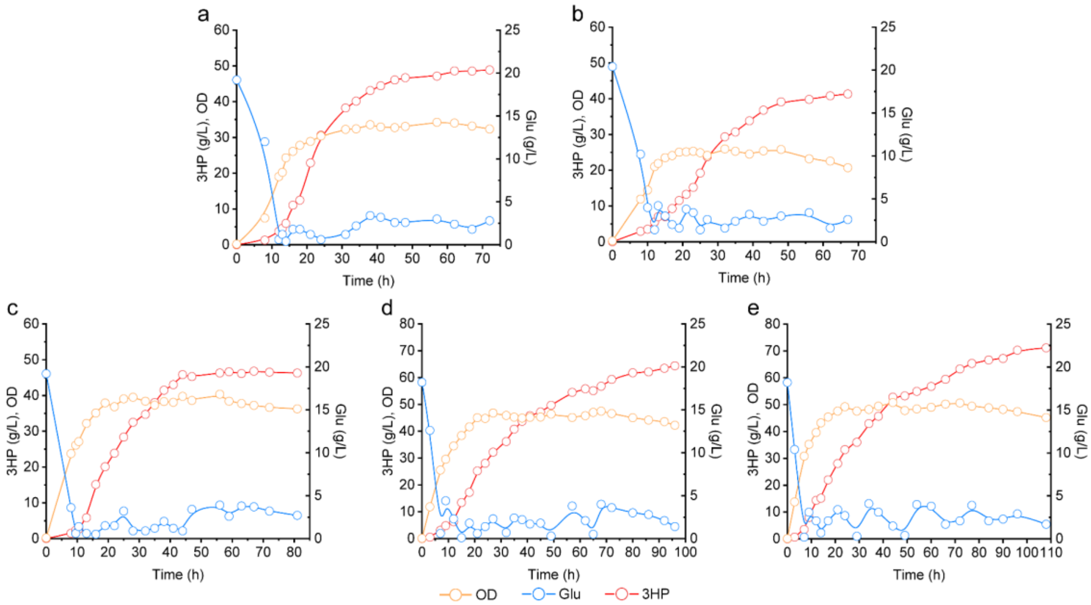

Supplementary Figure 22. 5-L bioreactor fermentation optimization for 3HP production. a. Fermentation medium composition: 9.8 g/L $K_2HPO_4 \cdot 3H_2O$, 2.1 g/L $C_6H_8O_7 \cdot H_2O$, 0.3 g/L ammonium ferric citrate, 0.25 g/L $MgSO_4 \cdot 7H_2O$, 5 g/L beef extract, 2% glucose and 1-mL of trace element solution (0.29 g/L $ZnSO_4 \cdot 7H_2O$, 0.37 g/L $(NH_4)_6Mo_7O_{24} \cdot 4H_2O$, 0.25 g/L $CuSO_4 \cdot 5H_2O$, 1.58 g/L $MnCl_2 \cdot 4H_2O$ and 2.47 g/L H_3BO_4). The 3HP final titer was 48.86 g/L with a yield of approximately 0.4653 g/g. **b**. Fermentation medium composition: 14 g/L $K_2HPO_4 \cdot 3H_2O$, 5.2 g/L KH_2PO_4, 1 g/L NaCl, 1 g/L NH_4Cl, 0.25 g/L $MgSO_4 \cdot 7H_2O$, 5 g/L yeast extract and 2% glucose. In the 5-L bioreactor, the strain grew relatively poorly under cultivation in this medium, with a maximum OD of roughly only 26, 75.8% of a. The final titer of 3HP was 41.32 g/L, with a yield of approximately 0.4184 g/g. After the experiments in a and b, it was confirmed that the medium composition in the 5-L bioreactor was still as in **a**. **c**. The composition of the supplement was changed. Unlike the only 50% glucose feeding in a, During the fed-batch cultivation, the cells were fed with a medium (50% glucose solution, 19.6 g/L $K_2HPO_4 \cdot 3H_2O$, 10 g/L beef extract, 0.5 g/L $MgSO_4 \cdot 7H_2O$ and 2-mL trace element solution). Following this way, however, the change in the composition of the supplement only improved the growth, but had no effect on the titer or yield. The aeration ratio of a, b and c was 0.5 vvm. **d**. The aeration ratio was increased to 1.5 vvm and the feeding composition remained as 50% glucose solution. The highest OD of the strain was 47.5, which was 1.39 times higher than a and 1.18 times higher than c. The final 3HP titer was 64.4 g/L and a yield of about 0.5366 g/g, indicating that the aeration ratio is very important for the 3HP fermentation process of the strain. **e**. The aeration ratio was further increased to 2.5 vvm. The results showed a significantly faster glucose consumption and growth rate of the strain, with a final 3HP production of 71.14 g/L and a yield of about 0.5283 g/g. Finally the aeration ratio was further increased to 3 vvm in Fig. 7f to further investigate the fermentation performance in the 5-L bioreactor. Source data are provided as a Source Data file.

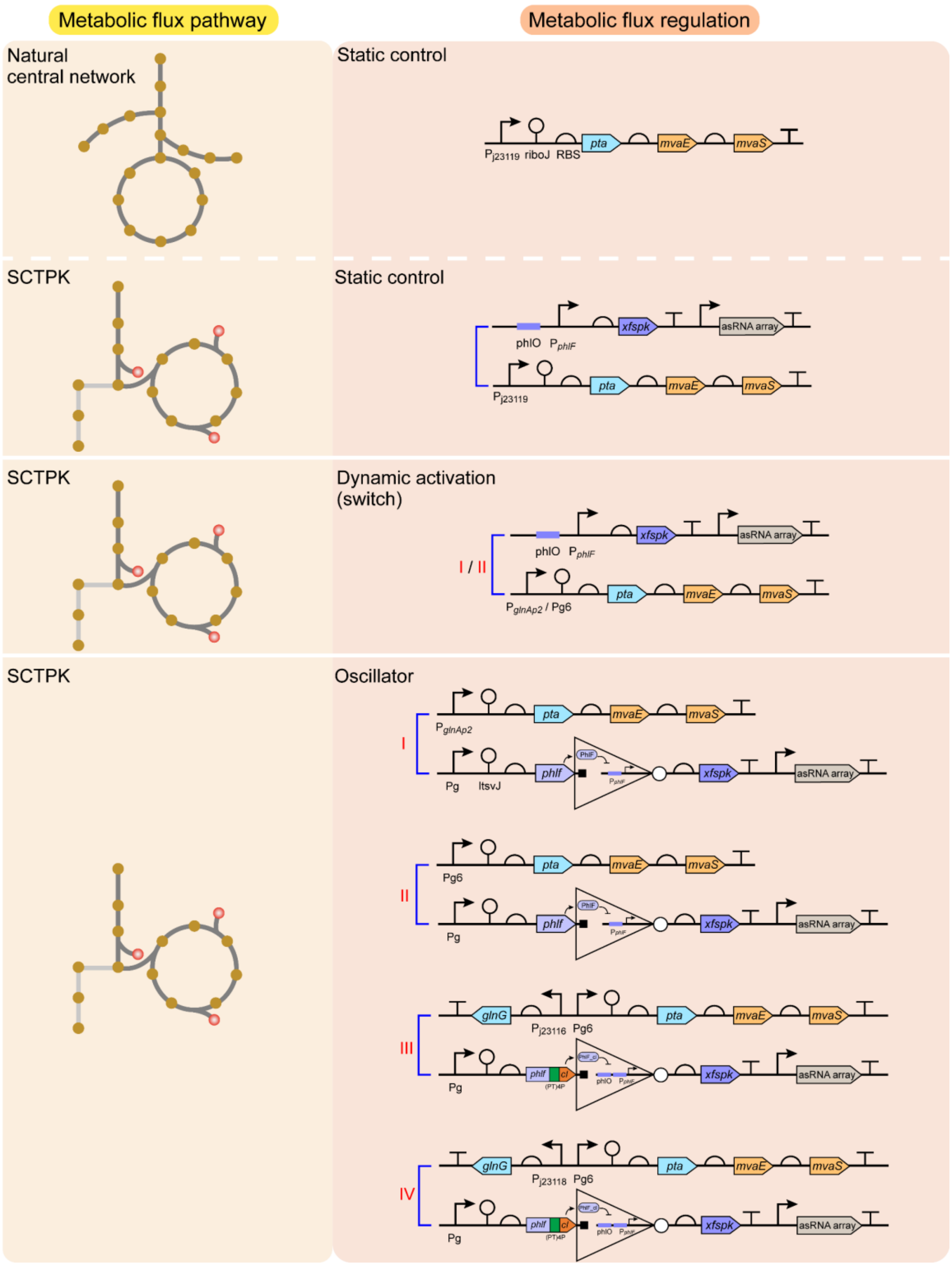

Supplementary Figure 23. Constructing different combinations of regulatory patterns for the biosynthesis of MVA. Among the metabolic flux pathways include the native central metabolic network and the SCTPK obtained by static reconfiguration. The regulation of metabolic flux includes static control, dynamic activation (switch) and oscillators. Genes related to MVA synthesis, including *pta*, *mvaE* and *mvaS*, were integrated in the downstream activation module. *pta*, phosphate acetyltransferase; *mvaE*, acetyl-CoA acetyltransferase/HMG-CoA reductase; *mvaS*, hydroxymethylglutaryl-CoA synthase.

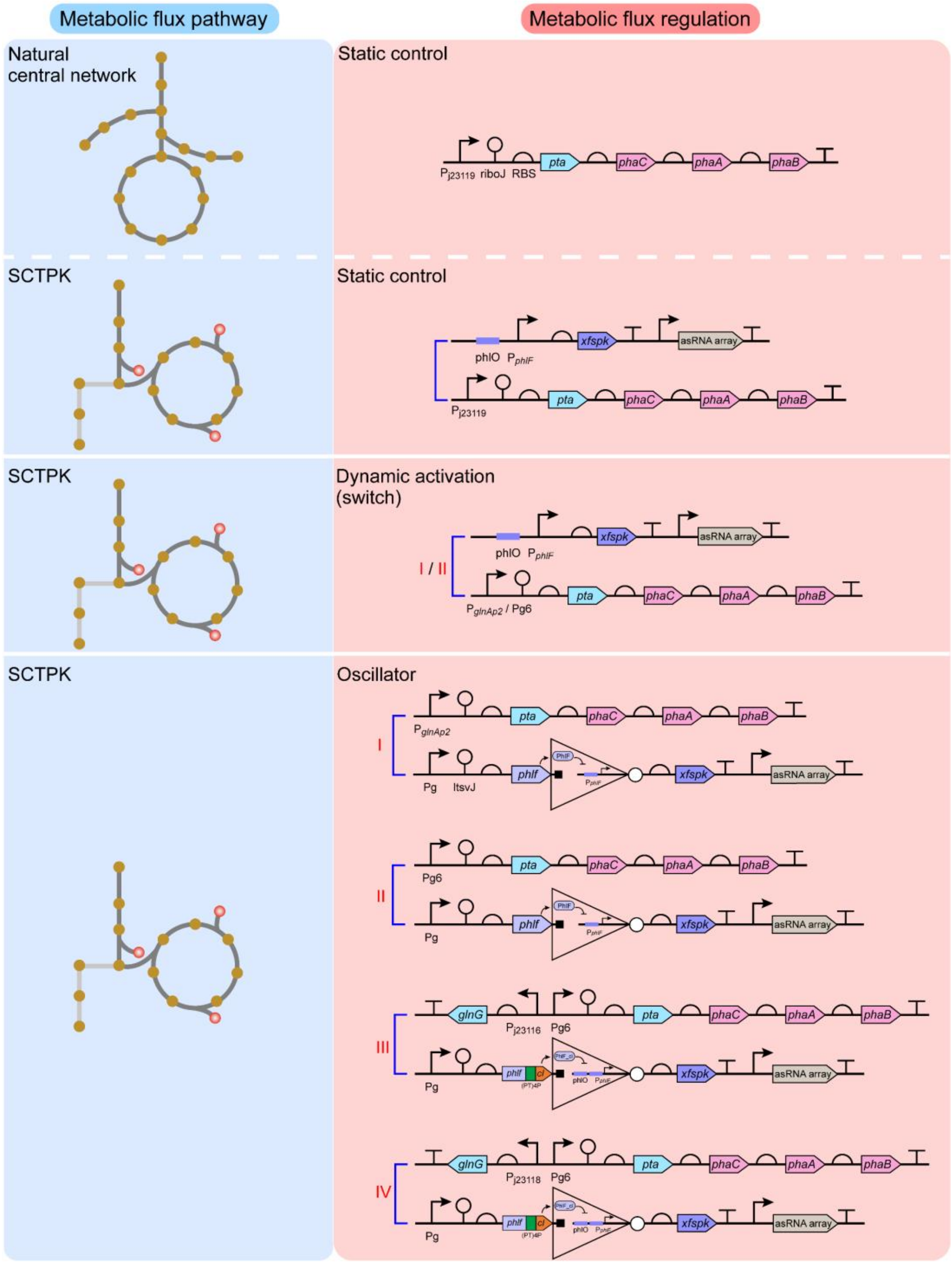

Supplementary Figure 24. Constructing different combinations of regulatory patterns for the biosynthesis of PHB. Among the metabolic flux pathways include the native central metabolic network and the SCTPK obtained by static reconfiguration. The regulation of metabolic flux includes static control, dynamic activation (switch) and oscillators. Genes related to PHB synthesis, including *pta*, *phaA, phaB* and *phaC*, were integrated in the downstream activation module. *pta*, phosphate acetyltransferase; *phaA*, acetyl-CoA acetyltransferase; *phaB*, 3-hydroxybutyryl-CoA dehydrogenase; *phaC*, 3-hydroxydecanoate polymerase.

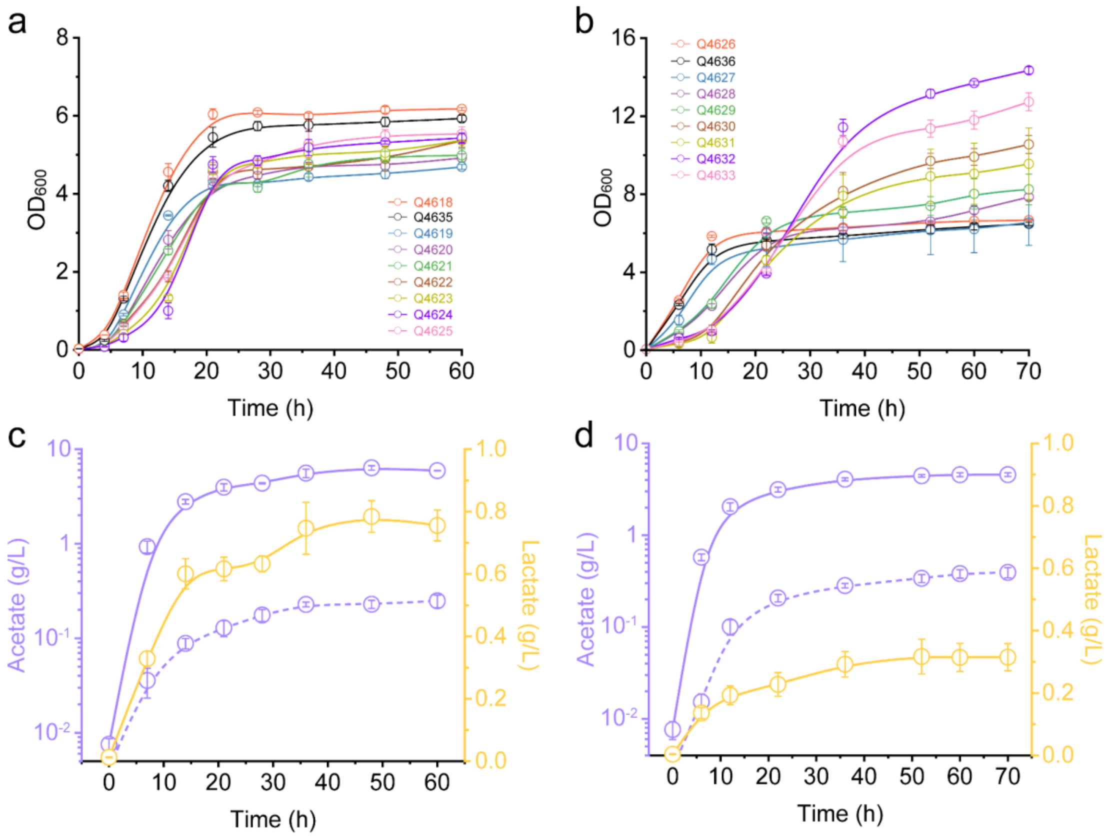

Supplementary Figure 25. Representative growth curves and byproducts generation of strains with different regulation patterns for MVA and PHB production during shake flask fermentation. These patterns include “static regulation”, “static regulation with SCTPK”, “SCTPK + Switch I / II” and “SCTPK + Oscillator I - IV”. **a**. **b**. The growth curves during MVA and PHB production, respectively. **c**. **d**. Detection of overflow byproducts during MVA (**c**) and PHB (**d**) fermentation. The solid lines represent the statically regulated strains (Q4618 and Q4626) carrying the native central metabolism. The dotted lines represent the strains carrying "SCTPK + Oscillator III for MVA" (Q4624) or "SCTPK + Oscillator III for PHB" (Q4632). All data were the average of three independent studies with standard deviations. Source data are provided as a Source Data file.

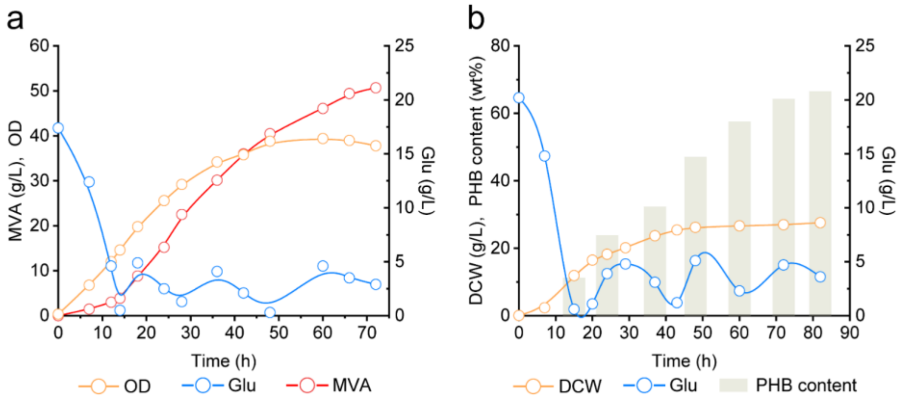

Supplementary Figure 26. Fed-batch cultures of strains carrying the pattern of "SCTPK + Oscillator III" in a 5-L bioreactor for the production of MVA and PHB, respectively. a. MVA fermentation; **b**. PHB fermentation. Source data are provided as a Source Data file.

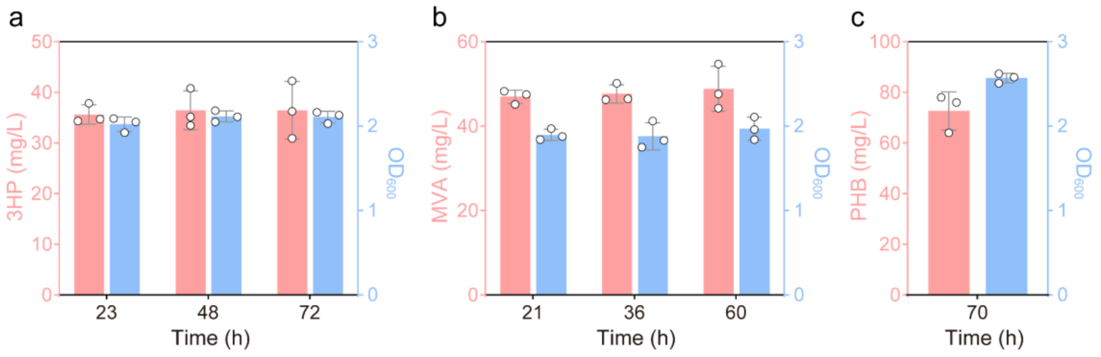

Supplementary Figure 27. Cell density and production of 3HP (a), MVA (b) and PHB (c) when grown in fermentation medium without glucose. Source data are provided as a Source Data file.

Supplementary references

1. Bogorad IW, Lin TS, Liao JC. Synthetic non-oxidative glycolysis enables complete carbon conservation. *Nature* **502**, 693-697 (2013).
2. Donahue JL, Bownas JL, Niehaus WG, Larson TJ. Purification and characterization of *glpX*-encoded fructose 1, 6-bisphosphatase, a new enzyme of the glycerol 3-phosphate regulon of *Escherichia coli*. *J Bacteriol* **182**, 5624-5627 (2000).
3. Wolf NM, Gutka HJ, Movahedzadeh F, Abad-Zapatero C. Structures of the *Mycobacterium tuberculosis* GlpX protein (class II fructose-1,6-bisphosphatase): implications for the active oligomeric state, catalytic mechanism and citrate inhibition. *Acta Crystallogr D Struct Biol* **74**, 321-331 (2018).
4. Woodruff WW, 3rd, Wolfenden R. Inhibition of ribose-5-phosphate isomerase by 4-phosphoerythronate. *J Biol Chem* **254**, 5866-5867 (1979).
5. Ueno-Nishio S, Mango S, Reitzer LJ, Magasanik B. Identification and regulation of the *glnL* operator-promoter of the complex *glnALG* operon of *Escherichia coli*. *J Bacteriol* **160**, 379-384 (1984).
