# Supplementary material for: Using a synthetic machinery to improve carbon yield with acetylphosphate as the core
Source: Nat Commun. 2023 Aug 30;14:5286. doi: 10.1038/s41467-023-41135-7 (PMC10468489; doi:10.1038/s41467-023-41135-7)
Supplement: Supplementary file 3 — Description of Additional Supplementary Files [file 41467_2023_41135_MOESM3_ESM.pdf]

Description of Additional Supplementary Files

File Name: Supplementary Data 1
Description: Characteristics of NOG and SCTPK pathways.

File Name: Supplementary Data 2
Description: Plasmids used in this study.

File Name: Supplementary Data 3
Description: Strains used in this study.

File Name: Supplementary Data 4
Description: Primers used in this study.

File Name: Supplementary Data 5
Description: Relevant input plasmid parts and their sequences.

File Name: Supplementary Data 6
Description: Relevant output plasmid parts and their sequences.

File Name: Supplementary Data 7
Description: The sedoheptulose-1,7-bisphosphatase from different strains used in this study.
