# Supplementary material for: Using a synthetic machinery to improve carbon yield with acetylphosphate as the core
Source: Nat Commun. 2023 Aug 30;14:5286. doi: 10.1038/s41467-023-41135-7 (PMC10468489; doi:10.1038/s41467-023-41135-7)
Supplement: Supplementary file 11 — Reporting Summary [file 41467_2023_41135_MOESM11_ESM.pdf]

nature portfolio

Corresponding author(s): Guang Zhao

Last updated by author(s): Aug 19, 2023

Reporting Summary

Nature Portfolio wishes to improve the reproducibility of the work that we publish. This form provides structure for consistency and transparency in reporting. For further information on Nature Portfolio policies, see our Editorial Policies and the Editorial Policy Checklist.

Statistics

For all statistical analyses, confirm that the following items are present in the figure legend, table legend, main text, or Methods section.

n/a	Confirmed	
☐	☒	The exact sample size (*n*) for each experimental group/condition, given as a discrete number and unit of measurement
☐	☒	A statement on whether measurements were taken from distinct samples or whether the same sample was measured repeatedly
☐	☒	The statistical test(s) used AND whether they are one- or two-sided<br>*Only common tests should be described solely by name; describe more complex techniques in the Methods section.*
☒	☐	A description of all covariates tested
☒	☐	A description of any assumptions or corrections, such as tests of normality and adjustment for multiple comparisons
☐	☒	A full description of the statistical parameters including central tendency (e.g. means) or other basic estimates (e.g. regression coefficient) AND variation (e.g. standard deviation) or associated estimates of uncertainty (e.g. confidence intervals)
☐	☒	For null hypothesis testing, the test statistic (e.g. F, t, r) with confidence intervals, effect sizes, degrees of freedom and P value noted<br>*Give P values as exact values whenever suitable.*
☒	☐	For Bayesian analysis, information on the choice of priors and Markov chain Monte Carlo settings
☒	☐	For hierarchical and complex designs, identification of the appropriate level for tests and full reporting of outcomes
☒	☐	Estimates of effect sizes (e.g. Cohen's *d*, Pearson's *r*), indicating how they were calculated

Our web collection on statistics for biologists contains articles on many of the points above.

Software and code

Policy information about availability of computer code

Data collection	Applied Biosystems QuantStudio 1 (Applied Biosystems) for qRT-PCR; Agilent HPLC 1260 with HPX-87H column (Bio-Rad, Hercules, CA) for metabolites analysis; SBA-40ES biological sensing analyzer (Institute of Biology, Shandong Academy of sciences, China) for glucose detection; Fusion FX6 Imaging System (Vilber, France) for protein signal detection; Laser Scanning Confocal Microscope LSM900 (ZEISS, Germany) for the fluorescence microscopy; A gas analysis system (BCP, BlueSens, Germany) for the detection of CO2 emissions; Multimode microplate reader (Spark, Tecan) for the assay of cell growth and fluorescence intensity.
Data analysis	Microsoft Excel 2019 was used to analyze the biomass, concentration and subsequent plotting were carried out using OriginPro 2022 (OriginLab) and Graphpad Prism 9 (Graphpad). The spacers of gRNA were designed by sgRNAcas9 v3.0. The fluorescence intensities were determined by using magellan 3.0 (Tecan). The imaging data were obtained and processed by Zen 3.3 (Zeiss) and Fiji 64 bit (NIH). Clustal Omega (https://www.ebi.ac.uk/Tools/msa/clustalo) was used for sequence alignment; RNAfold (http://rna.tbi.univie.ac.at/cgi-bin/RNAWebSuite/RNAfold.cgi) was used for structure predictions and Gibbs free energy calculations.

For manuscripts utilizing custom algorithms or software that are central to the research but not yet described in published literature, software must be made available to editors and reviewers. We strongly encourage code deposition in a community repository (e.g. GitHub). See the Nature Portfolio guidelines for submitting code & software for further information.

Data

Policy information about availability of data

All manuscripts must include a data availability statement. This statement should provide the following information, where applicable:

- Accession codes, unique identifiers, or web links for publicly available datasets
- A description of any restrictions on data availability
- For clinical datasets or third party data, please ensure that the statement adheres to our policy

Source data are provided with this paper. The sequences and accession numbers of used genes, the sequences of used primers, promoters, governors, enhancers, terminators, and linker peptides were provided in the Supplementary Information.

Research involving human participants, their data, or biological material

Policy information about studies with human participants or human data. See also policy information about sex, gender (identity/presentation), and sexual orientation and race, ethnicity and racism.

Reporting on sex and gender	N/A
Reporting on race, ethnicity, or other socially relevant groupings	N/A
Population characteristics	N/A
Recruitment	N/A
Ethics oversight	N/A

Note that full information on the approval of the study protocol must also be provided in the manuscript.

Field-specific reporting

Please select the one below that is the best fit for your research. If you are not sure, read the appropriate sections before making your selection.

☒ Life sciences ☐ Behavioural & social sciences ☐ Ecological, evolutionary & environmental sciences

For a reference copy of the document with all sections, see nature.com/documents/nr-reporting-summary-flat.pdf

Life sciences study design

All studies must disclose on these points even when the disclosure is negative.

Sample size	The strain growth assay, enzyme activity assay, fluorescence intensity assay, and concentration assay of multiple compounds including acetylphosphate, pyruvate, acetate, lactate, 3-hydroxypropionate, mevalonate, and polyhydroxybutyrate were performed at least three replicates. Two independent biological samples with three technical repeats for each sample were performed for each qRT-PCR analysis. Sample size was determined based on the previous experience and the majority of other metabolic engineering publications by Nature journals (e.g. Srinivasan et al Nature. 2020, Johnston et al Nature Communications. 2020). It is sufficient to confirm that results did not vary and were consistent.
Data exclusions	No data were excluded for the analyses.
Replication	All the biochemical and biological experiments were performed at least twice, with ability to obtain similar results.
Randomization	The samples of bacterial cultures that were split into different conditions were random samplings, and there is no control over which cells will be selected. And pipet tips were used to scoop few cells for culturing, the scooping locations are all random.
Blinding	Blinding is not relevant to our study because none of our data is based on qualitative scoring metrics nor does it involve animals or human research participants. As described in the above section for randomization, blinding during group allocation is irrelevant because the samples of bacterial cultures that were split into different conditions were random samplings and there is no control over which cells will be selected and thus, no bias during group allocation.

Reporting for specific materials, systems and methods

We require information from authors about some types of materials, experimental systems and methods used in many studies. Here, indicate whether each material, system or method listed is relevant to your study. If you are not sure if a list item applies to your research, read the appropriate section before selecting a response.

Materials & experimental systems

n/a	Involved in the study
☐	☒ Antibodies
☒	☐ Eukaryotic cell lines
☒	☐ Palaeontology and archaeology
☒	☐ Animals and other organisms
☒	☐ Clinical data
☒	☐ Dual use research of concern
☒	☐ Plants

Methods

n/a	Involved in the study
☒	☐ ChIP-seq
☒	☐ Flow cytometry
☒	☐ MRI-based neuroimaging

Antibodies

Antibodies used

Anti-6X His Tag antibody [GT359] (HRP), mouse monoclonal, Abcam catalog No. ab184607, 1:10000 diluted; Acetyl Lysine Mouse Monoclonal Antibody Catalog, EasyBio catalog No. BE3411, 1:2000 diluted; Goat Anti-Mouse IgG (H&L)-HRP Conjugated, EasyBio catalog No. BE0102, 1:10000 diluted.

Validation

Antibody was validated by manufacturers (WB, https://www.abcam.com/6x-his-tag-antibody-gt359-hrp-ab184607.html; http://www.bioeasytech.com/product/2654.html?goods_id=5194#; http://www.bioeasytech.com/product/2907.html?goods_id=5794#) and titrated in our laboratory prior to its use.
